# Supplementary material for: Rotator Cuff Tenocytes Differentiate into Hypertrophic Chondrocyte-Like Cells to Produce Calcium Deposits in an Alkaline Phosphatase-Dependent Manner
Source: J Clin Med. 2019 Sep 26;8(10):1544. doi: 10.3390/jcm8101544 (PMC6833470; doi:10.3390/jcm8101544)
Supplement: Supplementary file 1 [file jcm-08-01544-s001.pdf]

Supplementary material

Supplemental methods

Characterization of tenocytes like cells

Surface markers of TLC were determined at passage 2 by flow cytometry (FACS). The cells were incubated with antibodies against CD44, CD45, CD73, CD90, CD105, CD133 and CD146 for 20 minutes at room temperature. Unstained cells were used as controls (CT). After fixation, cells were analyzed with the BD-Accuri C6 Cytometer (BD Biosciences, Germany) and with BD-Accuri C6 Plus Software.

Table S1. Primers used for quantitative PCR.

GENE	**Forward**	**Reverse**
RUNT-RELATED TRANSCRIPTION FACTOR 2 (RUNX2)	gcctaggcgcatttcaga	gctcttcttactgagagtggaagg
SOX9	gtacccgcacttgcacaac	tcgctctcgttcagaagtctc
TYPE I COLLAGEN (COL1A1)	ctggacctaaaggtgctgct	gctccagcctctccatcttt
TYPE II COLLAGEN (COL2A1)	tggtgctaatggcgagaag	cccagtctctccacgttcac
TYPE III COLLAGEN (COL 3A1)	agggaaagatggcccaag	gcaccaccttcacccttatc
TYPE X COLLAGEN (COL10A1)	caccttctgcactgctcatc	ggcagcatattctcagatgga
SECRETED PHOSPHOPROTEIN 1 (SPP1)	gagggcttggttgtcagc	caattctcatggtagtgagttttcc
BONE GAMMA-CARBOXYGLUTAMATE PROTEIN (BGLAP)	ggcgctacctgtatcaatgg	tcagccaactcgtcacagtc
BONE SIALOPROTEIN (BSP)	cgaatacacgggcgtcaatg	gtagctgtactcatcttcataggc
TISSUE NON-SPECIFIC ALKALINE PHOPHATASE (TNAP)	aacaccacccaggggaac	ggtcacaatgcccacagatt
ECTONUCLEOTIDE PYROPHOSPHATE/PHOPHODIESTERASE 1 (ENPP1)	gaaagaccacacttttacactctg	ggctttgatgacttcactgct
MOHAWK HOMEOBOX (MKX)	gctcgcagatgacgctagt	tctggctgtcgaacggtatt
SCLERAXIS (SCX)	accttctgcctcagcaacca	ggccacctcctaactgcgaat
TENOMODULIN (TNMD)	gccagacaagcaagtgagga	ttgcctcgacggcagtaaat
CARTILAGE OLIGOMERIC MATRIX PROTEIN (COMP)	gacatcaggactctcgggacaac	tcgtcgtcgcaggcatcac
AGGRECAN (ACAN)	cccctgctatttcatcgaccc	gacacacggctccacttgat
MATRIX METALLOPEPTIDASE 13 (MMP13)	cctggacaagtagttccaaagg	gccggtgtaggtgtagatagga
GLYCERALDEHYDE-3-PHOSPHATE dehydrogenase (GAPDH)	tgggtgtgaaccatgagaagtatg	ggtgcaggaggcattgct

Supplemental figure S1. Histology and immunohistochemistry of a normal tendon (representative images). HE: haematoxylin and eosin; SO/FG: Safranin O Fast Green; COL2: type II collagen; ENPP1: Ectonucleotidepyrophosphatase/phosphodiesterase 1; TNAP: Tissue non-specific alkaline phosphatase; COL10: type X collagen. Scale bar = 200µM.

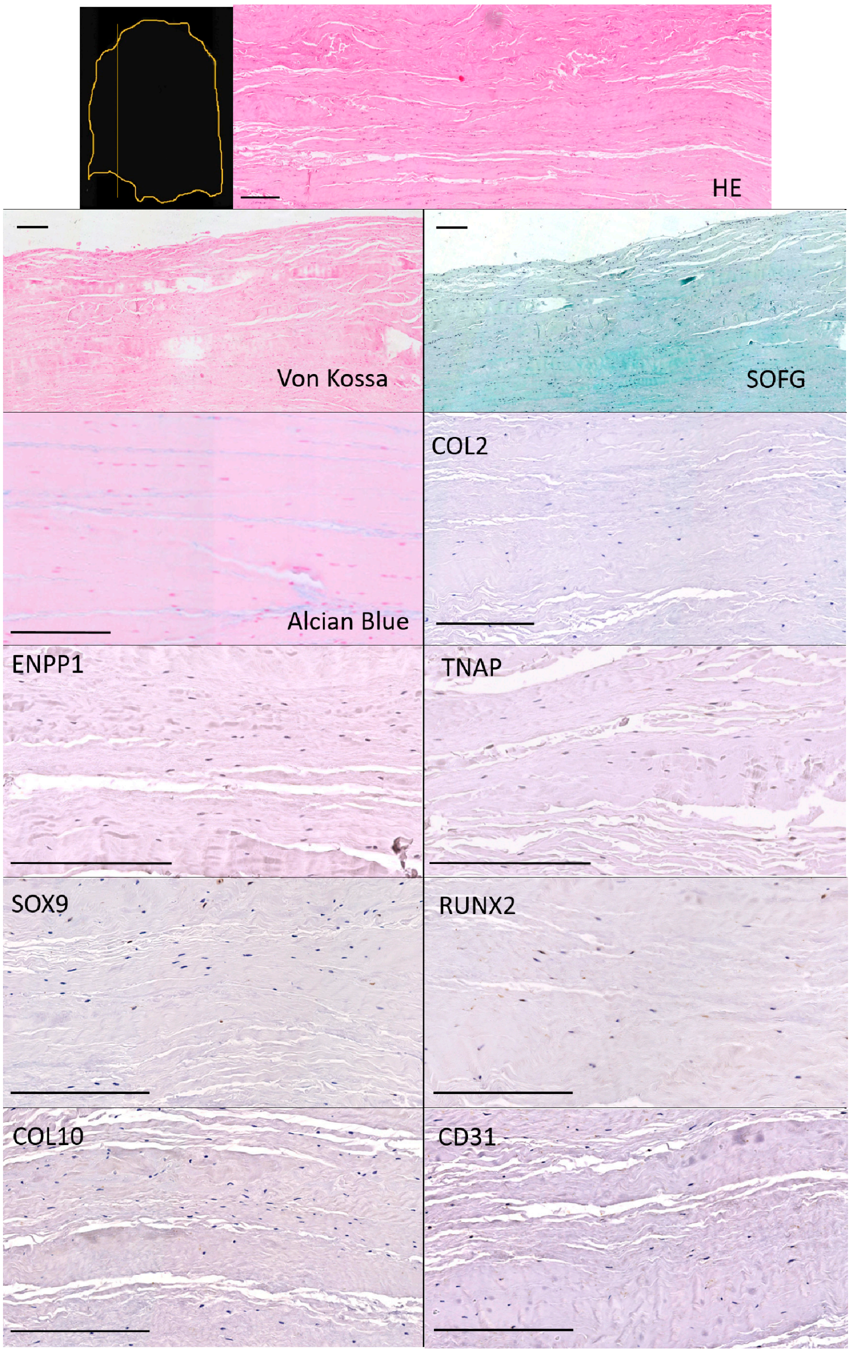

Supplemental figure S2. Characterization of tenocyte-like cells. A: Gene expression of tenocyte markers at different passages assessed by quantitative PCR (N=1). B: Surface markers of tenocyte-like cells assessed by Flow Cytometry (N=1). COL: collagen; MKX: Mohawk homeobox; SCX: scleraxis; TNMD: Tenomodulin; P: passage; ND: not detected; CT: control with unstained cells.

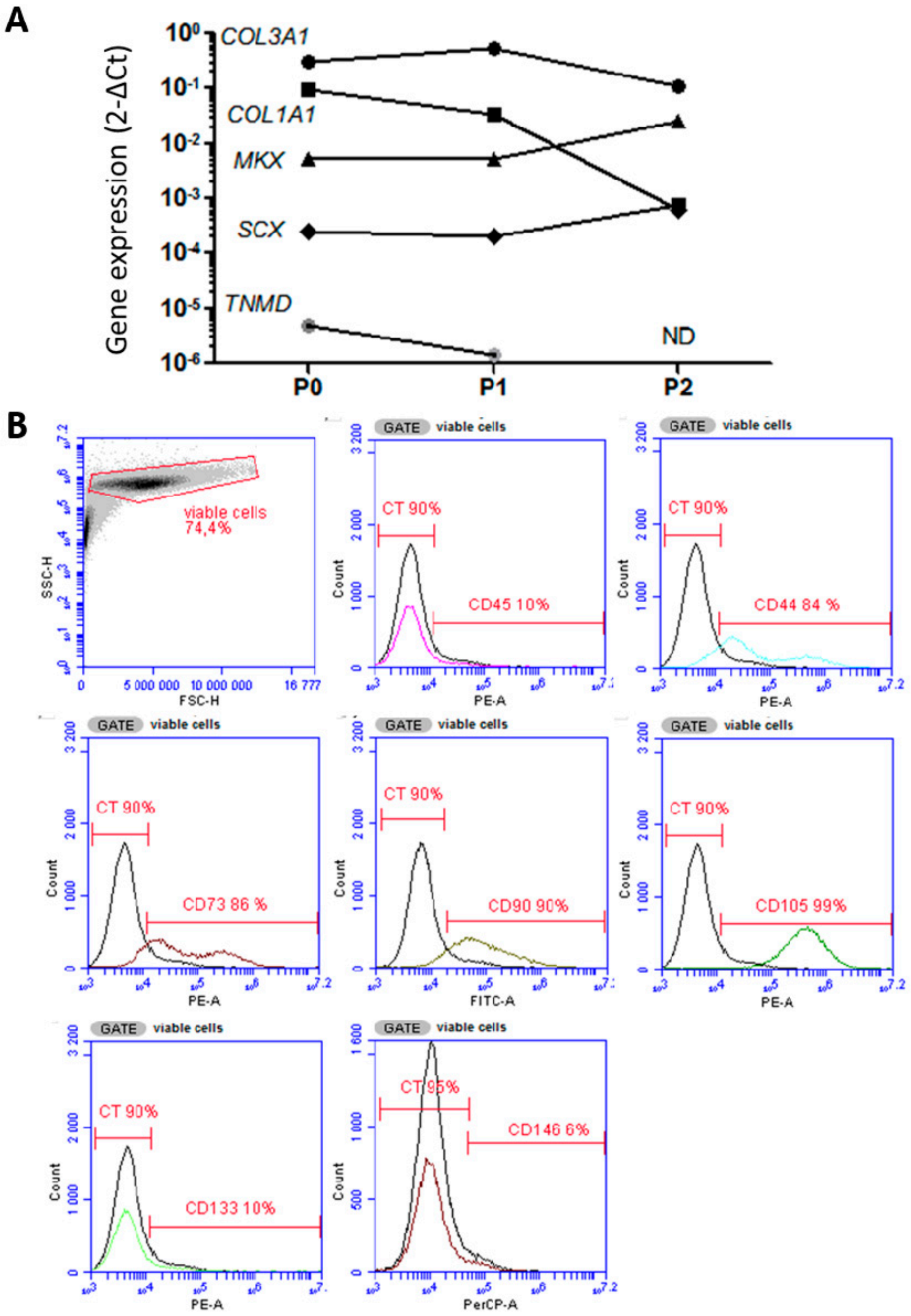

Supplementary Table S2. Proteomic analysis of calcium deposits from 5 patients.

P: peptides; SC: Spectral Count; BSC : Basic Spectral Count; SSC: Specific Spectral Count.

Accession	Description	Gene name	Patient 1			Patient 2			Patient 3			Patient 4			Patient 5			Total SSC
			P	BSC	SSC	P	BSC	SSC	P	BSC	SSC	P	BSC	SSC	P	BSC	SSC	
A0A024R462	Fibronectin 1, isoform CRA_n	FN1	72	136	136	63	110	110	74	132	132	87	221	221	75	169	169	768
CO3	Complement C3	C3	76	138	138	44	73	73	52	80	80	86	183	183	49	80	80	554
APOA1	Apolipoprotein A-I	APOA1	25	84	78	28	110	101	29	107	100	28	88	82	31	136	125	486
ANT3	Antithrombin-III	SERPINC1	27	60	60	21	38	38	26	53	53	26	68	68	24	49	49	268
PLMN	Plasminogen	PLG	32	40	40	24	29	29	29	38	38	39	64	64	38	63	63	234
CO6A3	Collagen alpha-3(VI) chain	COL6A3	30	36	36	19	22	22	27	39	39	50	64	64	32	39	39	200
APOA4	Apolipoprotein A-IV	APOA4	15	24	24	24	46	46	22	29	29	21	25	25	20	33	33	157
CLUS	Clusterin	CLU	12	21	21	12	27	27	13	28	28	21	42	42	19	36	36	154
GELS	Gelsolin	GSN	20	38	38	15	26	26	20	29	29	13	18	18	24	39	39	150
COMP	Cartilage oligomeric matrix protein	COMP	22	35	33	11	19	16	14	19	16	30	60	57	20	27	25	147
PRG4	Proteoglycan 4	PRG4	14	30	30	10	13	12	11	13	12	26	75	71	12	21	21	146
PEDF	Pigment epithelium-derived factor	SERPINF1	13	36	36	8	16	16	13	38	38	8	23	23	11	30	30	143
ALBU	Serum albumin	ALB	69	775	40	60	672	15	66	823	40	56	395	8	66	659	27	130
APOB	Apolipoprotein B-100	APOB	14	14	14	19	26	26	5	5	5	62	73	73	3	3	3	121
A0A024R884	Tenascin C (Hexabrachion), isoform CRA_a	TNC	17	20	20	8	10	10	13	14	14	39	47	47	21	25	25	116
AACT	Alpha-1-antichymotrypsin	SERPINA3	13	23	23	13	22	22	15	27	27	11	17	17	12	22	22	111
PCOC1	Procollagen C-endopeptidase enhancer 1	PCOLCE	12	20	20	12	18	18	14	21	21	13	25	25	13	25	25	109
B4DPP8	cDNA FLJ53075, highly similar to Kininogen-1		12	15	15	9	13	13	13	21	21	19	29	29	16	27	27	105
A0A384MDQ7	Epididymis secretory sperm binding protein		32	87	22	37	111	28	32	107	14	24	59	11	33	97	16	91
B4E1Z4	cDNA FLJ55673, highly similar to Complement factor B		15	19	19	12	13	13	14	16	16	15	19	19	13	18	18	85
CERU	Ceruloplasmin	CP	21	29	29	5	6	6	10	11	11	19	24	24	8	12	12	82
FGFP2	Fibroblast growth factor-binding protein 2	FGFBP2	11	16	16	9	16	16	9	16	16	11	17	17	12	16	16	81
VTNC	Vitronectin	VTN	5	10	10	7	9	9	5	8	8	14	41	41	6	11	11	79
CILP1	Cartilage intermediate layer protein 1	CILP	10	11	11	10	12	12	8	10	10	24	29	29	15	16	16	78
CO9	Complement component C9	C9	10	13	13	5	5	5	8	10	10	19	38	38	7	11	11	77
VTDB	Vitamin D-binding protein	GC	9	13	13	9	13	13	9	16	16	13	17	17	14	18	18	77
APOE	Apolipoprotein E	APOE	6	7	7	10	11	11	8	9	9	18	32	32	14	17	17	76
FIBB	Fibrinogen beta chain	FGB	11	13	13	8	11	11	11	13	13	28	35	35	3	3	3	75
THRB	Prothrombin	F2	12	15	15	3	5	5	5	8	8	21	37	37	6	9	9	74
F5GY80	Complement component C8 beta chain	C8B	11	17	17	6	10	10	6	8	8	16	19	19	11	12	12	66
FETUA	Alpha-2-HS-glycoprotein	AHSG	7	14	14	6	9	9	5	7	7	10	24	24	8	12	12	66
TENX	Tenascin-X	TNXB	8	8	8	14	15	15	12	13	13	14	14	14	12	12	12	62
A0A024R6I7	Alpha-1-antitrypsin	SERPINA1	29	69	4	33	87	4	31	112	19	25	59	11	35	101	20	58
CRAC1	Cartilage acidic protein 1	CRTAC1	1	12	12	7	7	7	5	5	5	1	21	21	8	10	10	55

			0									4						
Q6PIL8	IGK@ protein	IGK@	5	19	11	6	24	13	6	23	11	6	22	12	5	17	7	54
ALS	Insulin-like growth factor-binding protein complex acid labile subunit	IGFALS	9	12	12	5	5	5	7	7	7	12	17	17	7	12	12	53
LBP	Lipopolysaccharide-binding protein	LBP	9	12	12	2	3	3	5	6	6	8	17	17	7	13	13	51
HTRA1	Serine protease HTRA1	HTRA1	12	14	14	4	6	6	6	8	8	10	16	16	4	5	5	49
ANGT	Angiotensinogen	AGT	7	8	8	7	11	11	6	7	7	7	11	11	8	11	11	48
B4E1B2	cDNA FLJ53691, highly similar to Serotransferrin		4	5	5	8	8	8	10	12	12	10	13	13	9	10	10	48
TTHY	Transthyretin	TTR	8	12	12	4	5	5	8	14	14	9	9	9	8	8	8	48
CO8A	Complement component C8 alpha chain	C8A	8	10	10	4	5	5	5	5	5	16	18	18	8	9	9	47
CO8G	Complement component C8 gamma chain	C8G	4	10	10	2	5	5	3	6	6	8	15	15	6	11	11	47
FIBG	Fibrinogen gamma chain	FGG	8	8	8	5	6	6	7	7	7	17	20	20	3	3	3	44
CO1A2	Collagen alpha-2(I) chain	COL1A2	9	10	10	5	5	5	9	9	9	7	7	7	9	11	11	42
FIBA	Fibrinogen alpha chain	FGA	4	4	4	3	4	4	6	8	8	16	21	21	5	5	5	42
IPSP	Plasma serine protease inhibitor	SERPINA5	7	8	8	8	11	11	7	9	9	6	7	7	7	7	7	42
KAIN	Kallistatin	SERPINA4	8	11	11	5	6	6	6	7	7	7	9	9	7	9	9	42
SPRC	SPARC	SPARC	7	10	10	6	7	7	7	8	8	1	1	1	10	14	14	40
CO5	Complement C5	C5	5	5	5	6	7	7	1	1	1	19	22	22	4	4	4	39
ITIH4	Inter-alpha-trypsin inhibitor heavy chain H4	ITIH4	4	4	4	5	7	7	2	2	2	14	18	18	6	6	6	37
CO3A1	Collagen alpha-1(III) chain	COL3A1	4	4	4	5	6	6	6	9	9	5	6	6	10	11	11	36
HEP2	Heparin cofactor 2	SERPIND1	7	7	7	3	4	4	4	6	6	11	13	13	5	6	6	36
INHBA	Inhibin beta A chain	INHBA	3	3	3	3	4	4	6	6	6	8	9	9	11	14	14	36
Q6N093	Uncharacterized protein DKFZp686I04196 (Fragment)	DKFZp686I04196	10	18	10	7	10	2	13	25	9	12	25	10	10	18	5	36
APOA2	Apolipoprotein A-II	APOA2	3	7	7	5	7	7	5	7	7	4	8	8	3	6	6	35
Q6MZQ6	Uncharacterized protein DKFZp686G11190	DKFZp686G11190	12	25	5	11	22	7	13	31	7	12	32	8	13	31	7	34
APOC1	Apolipoprotein C-I	APOC1	3	3	3	4	9	9	4	6	6	5	8	8	3	6	6	32
BGH3	Transforming growth factor-beta-induced protein ig-h3	TGFBI	7	8	8	4	4	4	3	3	3	8	10	10	7	7	7	32
CCD80	Coiled-coil domain-containing protein 80	CCDC80	5	6	6	1	1	1	2	2	2	10	16	16	4	6	6	31
CD14	Monocyte differentiation antigen CD14	CD14	5	9	9	2	2	2	4	5	5	4	6	6	7	9	9	31
Q8NEJ1	Uncharacterized protein		3	7	4	4	9	4	5	16	9	5	9	6	4	11	8	31
CHAD	Chondroadherin	CHAD	6	10	10	4	5	5	1	1	1	6	10	10	3	4	4	30
HABP2	Hyaluronan-binding protein 2	HABP2	1	1	1	2	3	3	5	8	8	9	13	13	5	5	5	30
TSP1	Thrombospondin-1	THBS1	6	7	7	3	3	3	5	5	5	6	7	7	8	8	8	30
PRELP	Prolargin	PRELP	5	6	6	2	2	2	4	5	5	9	12	12	4	4	4	29
APOL1	Apolipoprotein L1	APOL1	2	2	2	3	4	4	4	4	4	8	10	10	7	8	8	28
CO7	Complement component C7	C7	3	3	3				3	3	3	14	18	18	4	4	4	28
LUM	Lumican	LUM	4	6	6	1	1	1	6	9	9	5	6	6	4	5	5	27
SAMP	Serum amyloid P-component	APCS	5	6	6	3	4	4	4	5	5	6	8	8	4	4	4	27
A2AP	Alpha-2-antiplasmin	SERPINF2	7	7	7	3	3	3	5	5	5	4	5	5	6	6	6	26
AMBP	Protein AMBP	AMBP	4	5	5	1	1	1	4	5	5	5	8	8	5	7	7	26
CO6A1	Collagen alpha-1(VI) chain	COL6A1	3	4	4	2	2	2	4	5	5	6	9	9	6	6	6	26
ENOA	Alpha-enolase	ENO1	5	6	6	3	3	3	4	4	4	9	10	10	2	3	3	26
MXRA5	Matrix-remodeling-associated protein 5	MXRA5	2	2	2							17	22	22	2	2	2	26
SODE	Extracellular superoxide dismutase [Cu-Zn]	SOD3	5	6	6	3	3	3	3	3	3	4	7	7	5	7	7	26
PGS2	Decorin	DCN	5	6	6	4	5	5	2	2	2	8	11	11	1	1	1	25
PON1	Serum paraoxonase/arylesterase 1	PON1	1	1	1	1	4	4	2	3	3	5	12	12	2	5	5	25
VIME	Vimentin	VIM				1	1	1	1	1	1	12	15	15	7	8	8	25

HBB	Hemoglobin subunit beta	HBB	7	12	4	3	4	2	7	11	4	5	6	2	11	22	12	24
G3P	Glyceraldehyde-3-phosphate dehydrogenase	GAPDH	3	4	4	3	3	3				8	9	9	5	7	7	23
FBLN1	Fibulin-1	FBLN1	2	2	2				1	1	1	12	16	16	3	3	3	22
ANXA1	Annexin A1	ANXA1	1	1	1	2	2	2	4	5	5	7	8	8	5	5	5	21
H14	Histone H1.4	HIST1H1E	4	5	5	2	3	3	2	3	3	3	4	4	4	6	6	21
HBA	Hemoglobin subunit alpha	HBA1	4	6	6	2	3	3	2	2	2	3	3	3	6	7	7	21
HPT	Haptoglobin	HP	5	6	4	3	4	3	9	11	7	5	7	6	2	3	1	21
IBP5	Insulin-like growth factor-binding protein 5	IGFBP5	3	3	3	1	3	3	3	4	4	6	7	7	4	4	4	21
LDHA	L-lactate dehydrogenase A chain	LDHA	3	3	3	1	1	1	1	1	1	10	14	14	2	2	2	21
PGRP2	N-acetylmuramoyl-L-alanine amidase	PGLYRP2	2	2	2							8	13	13	5	6	6	21
TARSH	Target of Nesh-SH3	ABI3BP	2	3	3	1	1	1	2	2	2	9	13	13	1	2	2	21
AEBP1	Adipocyte enhancer-binding protein 1	AEBP1	2	2	2							11	16	16	2	2	2	20
FHR1	Complement factor H-related protein 1	CFHR1	6	6	2	5	7	1	9	13	4	8	9	3	10	19	9	19
A0A024R498	Serpin peptidase inhibitor, clade E (Nexin, plasminogen activator inhibitor type 1), member 2, isoform CRA_b	SERPINE2	2	2	2							11	13	13	3	3	3	18
ANXA5	Annexin A5	ANXA5	4	4	4	1	1	1	4	4	4	7	7	7	2	2	2	18
CBPB2	Carboxypeptidase B2	CPB2	3	3	3	3	3	3	1	1	1	6	9	9	2	2	2	18
MMP3	Stromelysin-1	MMP3	1	1	1	1	1	1	4	4	4	7	8	8	4	4	4	18
APOD	Apolipoprotein D	APOD	1	1	1	3	4	4	2	2	2	6	6	6	4	4	4	17
CO6A2	Collagen alpha-2(VI) chain	COL6A2	2	2	2	1	1	1	3	3	3	7	9	9	2	2	2	17
FHR2	Complement factor H-related protein 2	CFHR2	5	5	3	4	5	2	7	11	6	4	5	2	7	8	4	17
PGK1	Phosphoglycerate kinase 1	PGK1	4	5	5							9	11	11	1	1	1	17
ANXA2	Annexin A2	ANXA2	3	3	3	1	2	2				6	7	7	4	4	4	16
CH3L1	Chitinase-3-like protein 1	CHI3L1	7	7	7	1	1	1	1	1	1	5	5	5	2	2	2	16
IC1	Plasma protease C1 inhibitor	SERPING1				3	5	5	2	2	2	7	7	7	2	2	2	16
IGK	Immunoglobulin kappa light chain		6	11	3	7	14	4	7	15	4	7	12	2	7	13	3	16
ITIH2	Inter-alpha-trypsin inhibitor heavy chain H2	ITIH2	1	1	1	1	1	1	2	2	2	8	10	10	2	2	2	16
MIME	Mimecan	OGN	4	6	6	2	2	2	2	3	3	2	3	3	2	2	2	16
TR11B	Tumor necrosis factor receptor superfamily member 11B	TNFRSF11B	3	4	4	1	1	1	3	3	3	2	3	3	5	5	5	16
A0A075B6Z2	T cell receptor alpha joining 56 (Fragment)	TRAJ56	1	4	4	1	5	5	1	3	3	1	2	2	1	1	1	15
B2R5G8	Serum amyloid A protein		4	4	1	4	5	1	3	3	1	5	15	8	5	9	4	15
TRFM	Melanotransferrin	MELTF				2	2	2	1	1	1	3	3	3	9	9	9	15
APOC3	Apolipoprotein C-III	APOC3	1	1	1	2	5	5	2	3	3	2	2	2	2	3	3	14
CILP2	Cartilage intermediate layer protein 2	CILP2	2	2	2	1	1	1	1	1	1	6	7	7	3	3	3	14
IGHG3	Immunoglobulin heavy constant gamma 3	IGHG3	8	15	3	9	11	2	12	21	4	11	22	3	9	18	2	14
LYSC	Lysozyme C	LYZ	2	2	2				3	3	3	3	4	4	5	5	5	14
MMP2	72 kDa type IV collagenase	MMP2	1	1	1							8	11	11	2	2	2	14
QSOX1	Sulfhydryl oxidase 1	QSOX1	2	2	2				1	1	1	6	6	6	5	5	5	14
A0A0X9UWL5	GCT-A5 light chain variable region (Fragment)		1	1	1	2	3	3	3	4	4	2	3	3	2	2	2	13
FNDC1	Fibronectin type III domain-containing protein 1	FNDC1	1	1	1				3	3	3	5	6	6	3	3	3	13
KPYM	Pyruvate kinase PKM	PKM										11	13	13				13
A0A0G2JPR0	Complement C4-A	C4A	29	37	3	12	15	1	6	7		45	71	5	17	20	3	12
A0A0X9USM3	GCT-A4 heavy chain variable region (Fragment)		2	3	3	3	4	3	2	3	2	3	3	1	4	5	3	12
FMOD	Fibromodulin	FMOD	1	1	1	1	1	1	2	2	2	2	5	5	2	3	3	12
RNAS4	Ribonuclease 4	RNASE4	1	1	1	1	1	1	2	2	2	2	3	3	3	5	5	12
APOH	Beta-2-glycoprotein 1	APOH				1	1	1	1	1	1	3	5	5	4	4	4	11
B1ALD9	Periostin	POSTN	8	8								26	37	7	13	17	4	11
CO4B	Complement C4-B	C4B	30	36	2	11	14		7	8	1	46	73	7	16	18	1	11
GPX3	Glutathione peroxidase 3	GPX3	1	1	1							6	8	8	2	2	2	11
ITIH1	Inter-alpha-trypsin inhibitor heavy chain H1	ITIH1	1	1	1	1	1	1	1	1	1	5	5	5	3	3	3	11

PGBM	Basement membrane-specific heparan sulfate proteoglycan core protein	HSPG2	6	7	1	2	2	1	7	10	1	14	14	7	7	12	1	11
THBG	Thyroxine-binding globulin	SERPINA7	2	2	2	3	3	3	3	3	3	1	1	1	2	2	2	11
A0N5G3	Rheumatoid factor G9 light chain (Fragment)	V-lambda-3				1	1	1	3	5	5	1	1	1	2	3	3	10
A2MG	Alpha-2-macroglobulin	A2M	3	3	3							7	7	7				10
AT2A2	Sarcoplasmic/endoplasmic reticulum calcium ATPase 2	ATP2A2							2	2					13	16	10	10
CCL18	C-C motif chemokine 18	CCL18	1	1	1	1	1	1	2	2	2	3	3	3	3	3	3	10
CO5A1	Collagen alpha-1(V) chain	COL5A1										7	9	9	1	1	1	10
CSPG2	Versican core protein	VCAN	1	1	1	2	2	2	1	1	1	5	6	6				10
TIMP2	Metalloproteinase inhibitor 2	TIMP2							3	3	3	2	2	2	3	5	5	10
ABCF1	ATP-binding cassette sub-family F member 1	ABCF1	1	3	3	1	2	2	1	2	2	1	1	1	1	1	1	9
ATPB	ATP synthase subunit beta, mitochondrial	ATP5F1B										2	2	2	7	7	7	9
F6KPG5	Albumin (Fragment)		68	737	2	59	660	3	65	786	3	53	387		63	633	1	9
KLKB1	Plasma kallikrein	KLKB1	1	1	1	1	1	1	2	2	2	5	5	5				9
L0R5A1	Alternative protein CSF2RB	CSF2RB	1	1	1	1	3	3	1	2	2	1	1	1	1	2	2	9
PCOC2	Procollagen C-endopeptidase enhancer 2	PCOLCE2	2	2	2	1	1	1	1	1	1	5	5	5				9
Q6NS95	IGL@ protein	IGL@	3	4	1	3	6	1	5	11	4	3	4	1	4	5	2	9
Q96K68	cDNA FLJ14473 fis, clone MAMMA1001080, highly similar to Homo sapiens SNC73 protein (SNC73) mRNA		3	6	2	3	4	2	4	6	2	6	6	2	4	4	1	9
SAA4	Serum amyloid A-4 protein	SAA4	4	4	1	3	4		3	3	1	6	10	3	6	9	4	9
A0A125QYY9	IBM-B2 heavy chain variable region (Fragment)		1	1	1	1	1	1	2	4	4	1	1	1	1	1	1	8
A2KBB9	Anti-(ED-B) scFV (Fragment)		3	4	2	4	4	1	4	4	1	5	6	2	5	6	2	8
ACTS	Actin, alpha skeletal muscle	ACTA1	2	2		4	4	1	4	4		6	11	2	10	13	5	8
AMPN	Aminopeptidase N	ANPEP										7	8	8				8
AT2A1	Sarcoplasmic/endoplasmic reticulum calcium ATPase 1	ATP2A1							5	5	3				9	11	5	8
B4DPR2	cDNA FLJ50830, highly similar to Serum albumin		53	626	7	42	552		48	660		38	315		47	523	1	8
ENPP1	Ectonucleotide pyrophosphatase/phosphodiesterase family member 1	ENPP1										8	8	8				8
HEMO	Hemopexin	HPX				1	1	1	1	1	1	3	3	3	2	3	3	8
PGS1	Biglycan	BGN	1	1	1							6	6	6	1	1	1	8
PLTP	Phospholipid transfer protein	PLTP	1	1	1	1	1	1				4	4	4	2	2	2	8
ZPI	Protein Z-dependent protease inhibitor	SERPINA10	2	2	2				1	1	1	3	5	5				8
1433Z	14-3-3 protein zeta/delta	YWHAZ	1	1		1	1	1	1	1	1	5	6	4	1	1	1	7
A0A0X9TD47	MS-D1 light chain variable region (Fragment)		1	1	1	1	1	1	2	2	2	1	2	2	1	1	1	7
C1S	Complement C1s subcomponent	C1S										5	5	5	1	2	2	7
CO2A1	Collagen alpha-1(II) chain	COL2A1				1	1	1	1	1	1	1	1	1	4	4	4	7
CO5A2	Collagen alpha-2(V) chain	COL5A2										5	6	6	1	1	1	7
D3DTX7	Collagen, type I, alpha 1, isoform CRA_a	COL1A1	3	5	5				1	1	1				1	1	1	7
MOES	Moesin	MSN	1	1	1							5	5	5	1	1	1	7
NUCB1	Nucleobindin-1	NUCB1	1	1	1				1	1	1	3	3	3	2	2	2	7
SEPP1	Selenoprotein P	SELENOP							3	3	3	2	2	2	2	2	2	7
TETN	Tetranectin	CLEC3B	1	1	1	1	1	1	2	2	2	1	1	1	2	2	2	7
TPIS	Triosephosphate isomerase	TPI1	1	1	1							3	6	6				7
APOF	Apolipoprotein F	APOF							1	1	1	2	3	3	1	2	2	6
CFAD	Complement factor D	CFD							2	2	2	1	1	1	2	3	3	6
COFA1	Collagen alpha-1(XV) chain	COL15A1										4	6	6				6
FA9	Coagulation factor IX	F9	2	2	2	1	1	1				2	2	2	1	1	1	6
IGD	Immunoglobulin delta heavy chain											4	4	4	2	2	2	6
IL11	Interleukin-11	IL11	2	2	2				2	2	2	1	1	1	1	1	1	6
LTBP1	Latent-transforming growth factor beta-binding protein 1	LTBP1										4	5	5	1	1	1	6

MATN2	Matrilin-2	MATN2	1	1	1							3	4	4	1	1	1	6
PAI1	Plasminogen activator inhibitor 1	SERPINE1										3	3	3	3	3	3	6
PLOD1	Procollagen-lysine,2-oxoglutarate 5-dioxygenase 1	PLOD1										5	6	6				6
PRDX1	Peroxiredoxin-1	PRDX1	1	1	1				1	1	1	3	3	3	1	1	1	6
Q9UL78	Myosin-reactive immunoglobulin light chain variable region (Fragment)		2	2	1	3	3	1	3	3	1	2	3	2	2	2	1	6
RET4	Retinol-binding protein 4	RBP4	1	1	1				2	2	2				2	3	3	6
TBA1A	Tubulin alpha-1A chain	TUBA1A	1	1	1	1	1	1				3	3	3	1	1	1	6
TSG6	Tumor necrosis factor-inducible gene 6 protein	TNFAIP6	1	1	1							3	3	3	2	2	2	6
TSP4	Thrombospondin-4	THBS4	2	2		3	3		1	3		8	9	5	3	3	1	6
VDAC1	Voltage-dependent anion-selective channel protein 1	VDAC1							1	1	1	1	1	1	4	4	4	6
5NTD	5'-nucleotidase	NT5E										5	5	5				5
A0A1B1CYC8	Vitamin D binding protein (Fragment)	Gc	1	1	1	1	2	2	1	1	1	1	1	1				5
ACTN4	Alpha-actinin-4	ACTN4	1	1								11	11	5				5
ANGI	Angiogenin	ANG							1	1	1	2	2	2	2	2	2	5
C1R	Complement C1r subcomponent	C1R										5	5	5				5
CADH1	Cadherin-1	CDH1	1	1	1							3	3	3	1	1	1	5
CAMP	Cathelicidin antimicrobial peptide	CAMP	1	1	1				1	1	1	1	1	1	1	2	2	5
CD109	CD109 antigen	CD109										5	5	5				5
DAF	Complement decay-accelerating factor	CD55				1	1	1				2	2	2	2	2	2	5
DKK3	Dickkopf-related protein 3	DKK3							1	1	1	1	1	1	3	3	3	5
EF1A1	Elongation factor 1-alpha 1	EEF1A1	1	1	1							3	3	3	1	1	1	5
H4	Histone H4	HIST1H4A	1	1	1				1	1	1	2	2	2	1	1	1	5
HV349	Immunoglobulin heavy variable 3-49	IGHV3-49	1	1	1	1	1	1				2	3	3				5
IGHG4	Immunoglobulin heavy constant gamma 4	IGHG4	6	11		5	6		9	16	3	8	14	2	6	12		5
ITB1	Integrin beta-1	ITGB1										5	5	5				5
LTBP2	Latent-transforming growth factor beta-binding protein 2	LTBP2										4	5	5				5
SAP	Prosaposin	PSAP										2	3	3	1	2	2	5
THY1	Thy-1 membrane glycoprotein	THY1				2	2	2	1	1	1				2	2	2	5
TIMP1	Metalloproteinase inhibitor 1	TIMP1	1	1	1	1	1	1	1	1	1	1	1	1	1	1	1	5
TIMP3	Metalloproteinase inhibitor 3	TIMP3										5	5	5				5
TM198	Transmembrane protein 198	TMEM198	1	1	1	1	1	1	2	2	2				1	1	1	5
A0A120HG46	GCT-A10 heavy chain variable region (Fragment)		2	2	2	1	1	1	1	1	1				1	1		4
A0A2P9AAK1	ATP-dependent DNA ligase	BQ8482_110076	1	1	1							1	1	1	1	2	2	4
ACTG	Actin, cytoplasmic 2	ACTG1	5	5		3	3		4	4		13	20	4	7	10		4
ADT1	ADP/ATP translocase 1	SLC25A4													2	4	4	4
C1QC	Complement C1q subcomponent subunit C	C1QC										1	2	2	2	2	2	4
CATD	Cathepsin D	CTSD										4	4	4				4
CFAH	Complement factor H	CFH	29	34		29	35		36	46	2	30	34		36	48	2	4
CO6	Complement component C6	C6	9	10		6	6		13	15	1	10	14	2	10	12	1	4
COCA1	Collagen alpha-1(XII) chain	COL12A1										1	1	1	3	3	3	4
COEA1	Collagen alpha-1(XIV) chain	COL14A1										3	4	4				4
COIA1	Collagen alpha-1(XVIII) chain	COL18A1	1	1	1				1	1	1	1	1	1	1	1	1	4
CRP	C-reactive protein	CRP	1	1	1				1	1	1	2	2	2				4
FSTL1	Follistatin-related protein 1	FSTL1				1	1	1	1	1	1	1	1	1	1	1	1	4
GDIR1	Rho GDP-dissociation inhibitor 1	ARHGDIA	1	1	1				1	1	1	1	1	1	1	1	1	4
GSTP1	Glutathione S-transferase P	GSTP1	1	1	1							2	2	2	1	1	1	4
GTR1	Solute carrier family 2, facilitated glucose transporter	SLC2A1	1	1	1							1	1	1	2	2	2	4

	member 1																	
HBD	Hemoglobin subunit delta	HBD	2	3					1	1		1	1		7	10	4	4
HPTR	Haptoglobin-related protein	HPR	2	2		1	1		4	4		3	3	2	3	4	2	4
IGHM	Immunoglobulin heavy constant mu	IGHM	1	1	1	1	1	1	3	3	1	5	5	1				4
LEG1	Galectin-1	LGALS1										4	4	4				4
SPON1	Spondin-1	SPON1										3	3	3	1	1	1	4
UGDH	UDP-glucose 6-dehydrogenase	UGDH										4	4	4				4
1433G	14-3-3 protein gamma	YWHAG	1	1								4	5	3				3
A0A024RC55	Milk fat globule-EGF factor 8 protein, isoform CRA_a	MFGE8	5	6		4	5		5	5		15	21	3	7	10		3
A0A024RDT5	Periostin, osteoblast specific factor, isoform CRA_a	POSTN	8	8								23	32	2	10	14	1	3
A0A193CHR0	10E8 heavy chain variable region (Fragment)		1	1		1	1		1	1		3	3	1	4	4	2	3
A0A2U8J8J2	Ig heavy chain variable region (Fragment)	IgH	1	1	1				1	1	1	1	1	1	1	1		3
A1AG1	Alpha-1-acid glycoprotein 1	ORM1							1	1	1	2	2	2				3
A1BG	Alpha-1B-glycoprotein	A1BG										2	3	3				3
A2J1M8	Rheumatoid factor RF-IP12 (Fragment)		1	1	1							1	1	1	1	1	1	3
AFAM	Afamin	AFM							2	2	2				1	1	1	3
ANXA4	Annexin A4	ANXA4										3	3	3				3
APOM	Apolipoprotein M	APOM	4	4	2	1	1		2	2		4	7		3	4	1	3
ASPN	Asporin	ASPN										3	3	3				3
ATPA	ATP synthase subunit alpha, mitochondrial	ATP5F1A										1	1	1	2	2	2	3
BASI	Basigin	BSG										3	3	3				3
CALM1	Calmodulin-1	CALM1				1	1	1	1	2	2							3
CD5L	CD5 antigen-like	CD5L							2	2	2	1	1	1				3
CD81	CD81 antigen	CD81										3	3	3				3
CFAI	Complement factor I	CFI										1	1	1	1	2	2	3
CH3L2	Chitinase-3-like protein 2	CHI3L2										3	3	3				3
CXL13	C-X-C motif chemokine 13	CXCL13										2	3	3				3
DSG1	Desmoglein-1	DSG1				2	2	2	1	1	1							3
FBLN3	EGF-containing fibulin-like extracellular matrix protein 1	EFEMP1										2	2	2	1	1	1	3
HV374	Immunoglobulin heavy variable 3-74	IGHV3-74	2	2	1	1	1		1	1		3	3	1	3	3	1	3
IBP3	Insulin-like growth factor-binding protein 3	IGFBP3										1	2	2	1	1	1	3
IQGA1	Ras GTPase-activating-like protein IQGAP1	IQGAP1										3	3	3				3
ITA5	Integrin alpha-5	ITGA5										3	3	3				3
ITAV	Integrin alpha-V	ITGAV										2	3	3				3
KAD1	Adenylate kinase isoenzyme 1	AK1										2	3	3				3
OAF	Out at first protein homolog	OAF	1	1	1							1	1	1	1	1	1	3
PLAK	Junction plakoglobin	JUP													3	3	3	3
PPIC	Peptidyl-prolyl cis-trans isomerase C	PPIC										1	2	2	1	1	1	3
Q59EG0	Basement membrane-specific heparan sulfate proteoglycan core protein variant (Fragment)		6	7	1	1	1		7	10	1	7	7		7	12	1	3
Q9UL83	Myosin-reactive immunoglobulin light chain variable region (Fragment)					3	4	1	1	2	2	1	1		1	2		3
RL40	Ubiquitin-60S ribosomal protein L40	UBA52				1	2	2							1	1	1	3
S10A7	Protein S100-A7	S100A7													2	3	3	3
S29A1	Equilibrative nucleoside transporter 1	SLC29A1										3	3	3				3
S6BGE0	IgG H chain		8	16	1	6	12	1	6	16		6	19		8	19	1	3
SRPX2	Sushi repeat-containing protein SRPX2	SRPX2	1	1	1							2	2	2				3
TRY1	Trypsin-1	PRSS1				1	1	1				1	1	1	1	1	1	3
TRY3	Trypsin-3	PRSS3	1	1	1	1	1	1	1	1	1							3
A0A0X9UWK7	MS-D4 heavy chain variable region (Fragment)											2	2	1	1	1	1	2
A0A0X9V9B3	MS-F1 light chain variable region (Fragment)								1	1	1	1	1	1				2
A0A1L2BU40	Anti-staphylococcal enterotoxin E variable region lambda chain (Fragment)														1	2	2	2
A0A2P9A	3-hydroxyisobutyrate dehydrogenase	BQ8482										1	1	1	1	1	1	2

XT4		_90344																
A0A2R8Y4P5	Required for meiotic nuclear division protein 1 homolog (Fragment)	RMND1													1	2	2	2
A0A2U8J8Q6	Ig heavy chain variable region (Fragment)	IgH				1	1	1				1	1	1				2
A0A2U8J8T1	Ig heavy chain variable region (Fragment)	IgH	1	1	1							2	2	1	1	1		2
A0A2U8J936	Ig heavy chain variable region (Fragment)	IgH										1	1	1	1	1	1	2
A2NB45	Cold agglutinin FS-1 L-chain (Fragment)								2	2	2							2
A8K5T0	cDNA FLJ75416, highly similar to Homo sapiens complement factor H (CFH), mRNA		29	34		31	37	2	35	44		30	34		34	46		2
A8K8Z4	cDNA FLJ78071, highly similar to Human MHC class III complement component C6 mRNA		10	11	1	6	6		12	14		9	12		10	12	1	2
AAAT	Neutral amino acid transporter B(0)	SLC1A5										1	2	2				2
ACTB	Actin, cytoplasmic 1	ACTB	5	5		3	3		4	4		12	17	1	8	11	1	2
ACTN1	Alpha-actinin-1	ACTN1	1	1								8	8	2				2
ANGL2	Angiopoietin-related protein 2	ANGPTL2										2	2	2				2
ANXA6	Annexin A6	ANXA6										2	2	2				2
AQP1	Aquaporin-1	AQP1										2	2	2				2
ARF1	ADP-ribosylation factor 1	ARF1										2	2	1	1	1	1	2
B2MG	Beta-2-microglobulin	B2M							1	2	2							2
B2R8I2	cDNA, FLJ93914, highly similar to Homo sapiens histidine-rich glycoprotein (HRG), mRNA		10	20	1	6	6	1	8	15		13	22		11	21		2
B4DJ26	Phosphoribosylformylglycinamidine synthase (FGAR amidotransferase), isoform CRA_c	PFAS				1	2	2										2
C1QB	Complement C1q subcomponent subunit B	C1QB	1	1	1	1	1	1										2
C4BPA	C4b-binding protein alpha chain	C4BPA										2	2	2				2
CD9	CD9 antigen	CD9										2	2	2				2
CLC11	C-type lectin domain family 11 member A	CLEC11A										2	2	2				2
CLIC1	Chloride intracellular channel protein 1	CLIC1										2	2	2				2
CLIC4	Chloride intracellular channel protein 4	CLIC4										2	2	2				2
CNTRL	Centriolin	CNTRL										1	1	1	1	1	1	2
COF1	Cofilin-1	CFL1	1	1	1										1	1	1	2
CSPG4	Chondroitin sulfate proteoglycan 4	CSPG4										2	2	2				2
CYC	Cytochrome c	CYCS	1	1	1				1	1	1							2
CYTC	Cystatin-C	CST3	1	1	1				1	1	1							2
EF2	Elongation factor 2	EEF2										2	2	2				2
FA10	Coagulation factor X	F10	1	1	1	1	1	1										2
FABPH	Fatty acid-binding protein, heart	FABP3										1	1	1	1	1	1	2
FBN1	Fibrillin-1	FBN1										1	1	1	1	1	1	2
FHR3	Complement factor H-related protein 3	CFHR3	2	2		1	1		1	1		1	1	1	3	3	1	2
FILA2	Filaggrin-2	FLG2													1	2	2	2
GDIB	Rab GDP dissociation inhibitor beta	GDI2										2	2	2				2
H2B1B	Histone H2B type 1-B	HIST1H2BB				1	1	1				1	1	1				2
HRG	Histidine-rich glycoprotein	HRG	10	20	1	5	5		8	15		14	23	1	11	21		2
HSPB1	Heat shock protein beta-1	HSPB1										1	1	1	1	1	1	2
IGF2	Insulin-like growth factor II	IGF2										2	2	2				2
ISK5	Serine protease inhibitor Kazal-type 5	SPINK5										1	2	2				2
KPRP	Keratinocyte proline-rich protein	KPRP				1	1	1							1	1	1	2
MA1A1	Mannosyl-oligosaccharide 1,2-alpha-mannosidase IA	MAN1A1										2	2	2				2
MAMC2	MAM domain-containing protein 2	MAMDC2										1	2	2				2
MED30	Mediator of RNA polymerase II transcription subunit 30	MED30										1	1	1	1	1	1	2
MGST3	Microsomal glutathione S-transferase 3	MGST3										1	1	1	1	1	1	2
MMRN2	Multimerin-2	MMRN2							1	1	1				1	1	1	2
MRCKB	Serine/threonine-protein kinase MRCK beta	CDC42BPB	1	1	1							1	1	1				2

NB5R3	NADH-cytochrome b5 reductase 3	CYB5R3										2	2	2				2
NBL1	Neuroblastoma suppressor of tumorigenicity 1	NBL1										1	2	2				2
NEUS	Neuroserpin	SERPINI1													2	2	2	2
PA2GA	Phospholipase A2, membrane associated	PLA2G2A	1	1	1							1	1	1				2
PGCA	Aggrecan core protein	ACAN										2	2	2				2
PLOD2	Procollagen-lysine,2-oxoglutarate 5-dioxygenase 2	PLOD2										2	2	2				2
PLSL	Plastin-2	LCP1	1	1	1							3	3	1				2
PLST	Plastin-3	PLS3										4	4	2				2
PPIB	Peptidyl-prolyl cis-trans isomerase B	PPIB	1	1	1							1	1	1				2
Q6U2L6	C4B (Fragment)	C4B	1	1	1										1	1	1	2
Q9UL85	Myosin-reactive immunoglobulin kappa chain variable region (Fragment)					2	3					2	2	1	2	3	1	2
Q9Y355	Apolipoprotein A1 (Fragment)		4	6		6	10	1	4	7		6	7	1	4	11		2
QCR2	Cytochrome b-c1 complex subunit 2, mitochondrial	UQCRC2													1	2	2	2
S10A8	Protein S100-A8	S100A8													2	2	2	2
S6BAM6	IgG H chain		7	15		5	11		6	16		7	20	1	8	19	1	2
SEM3C	Semaphorin-3C	SEMA3C										1	1	1	1	1	1	2
SFRP2	Secreted frizzled-related protein 2	SFRP2										2	2	2				2
SIL1	Nucleotide exchange factor SIL1	SIL1										2	2	2				2
SRCRL	Soluble scavenger receptor cysteine-rich domain-containing protein SSC5D	SSC5D										1	1	1	1	1	1	2
SRPX	Sushi repeat-containing protein SRPX	SRPX										2	2	2				2
TAGL2	Transgelin-2	TAGLN2										2	2	2				2
TBB2A	Tubulin beta-2A chain	TUBB2A										2	2	2				2
TITIN	Titin	TTN										1	1	1	1	1	1	2
TLN1	Talin-1	TLN1										2	2	2				2
TM109	Transmembrane protein 109	TMEM109										1	1	1	1	1	1	2
TUT7	Terminal uridylyltransferase 7	TUT7							1	1	1	1	1	1				2
VAT1	Synaptic vesicle membrane protein VAT-1 homolog	VAT1										2	2	2				2
VINC	Vinculin	VCL										2	2	2				2
WISP2	WNT1-inducible-signaling pathway protein 2	WISP2													2	2	2	2
